# Equilibrium Values for the Si-H Bond Length and Equilibrium Structures of Silyl Iodide and Halosilylenes

**DOI:** 10.3390/molecules29133101

**Published:** 2024-06-28

**Authors:** Jean Demaison, Jacques Liévin

**Affiliations:** 1 Physique des Lasers, Atomes et Molécules, Université de Lille, Bât. P5, 59655 Villeneuve d’Ascq cedex, France; 2 Spectroscopy, Quantum Chemistry and Atmospheric Remote Sensing (SQUARES), CP160/09, Faculté des Sciences, Université Libre de Bruxelles (U.L.B.), Ave. Roosevelt, 50, B-1050 Brussels, Belgium; jacky.lievin@ulb.be

**Keywords:** equilibrium structure, Si-H bond, silyl halides, silylene halides, ab initio

## Abstract

The equilibrium structures of silyl iodide, SiH_3_I, and silylene halides, SiHX (X = F, Cl, Br, I), were determined by using the mixed regression method, where approximate values of the rotational constants are supplemented by the structural parameters of a different origin. For this goal, it is shown that the r(Si-H) bond length can be determined by using the isolated SiH stretching frequency and that an accurate estimation of the bond angles is obtained by an MP2 calculation with a basis set of triple zeta quality. To check the accuracy of the experimental structures, they were also optimized by means of all electron CCSD(T) calculations using basis sets of quadruple zeta quality.

## 1. Introduction

Equilibrium structures are known for silyl fluoride, SiH_3_F [1], silyl chloride, SiH_3_Cl [2], silyl bromide, SiH_3_Br [3], SiH_3_CN [4], and silane, SiH_4_ [5]. It is not easy to determine an accurate equilibrium structure (corresponding to the minimum of the potential hypersurface) because, in addition to the ground state rotational constants, it requires the knowledge of at least the vibration–rotation interaction constants of all fundamental vibrational states or, in other words, the rotational constants of all fundamental vibrational states. This is often a difficult problem because some of these vibrational states are perturbed by nearby states (through anharmonic resonances or Coriolis interactions) and reliable analysis of the corresponding rovibrational spectra is not straightforward. This is particularly true for a molecule such as silyl iodide, SiH_3_I, whose rovibrational spectrum is complicated by the large quadrupole hyperfine structure due to the iodine nucleus. This is furthermore complicated by the small value of the *B* rotational constant, making the rovibrational spectrum very dense. For these reasons, approximate structures are often obtained by empirical methods. This is the case for SiH_3_I for which only empirical effective structures (*r*_0_) are available [6,7]. For this kind of empirical structure, all the rovibrational effects are neglected assuming that the equilibrium rotational constants are identical to the ground state rotational constants. These two *r*_0_-structures are significantly different and none of them is expected to be close to the equilibrium structure. This is unfortunate because no equilibrium value is known for the Si-I bond length. Actually, a *r*_0_ structure is available for monoiodosilylene, HSiI [8,9], but it is not expected to be a good approximation of the equilibrium structure.

The goal of this work is to present a set of consistent and accurate values for the Si-H bond length and to determine the equilibrium structure of SiH_3_I and of the silylene halides, HSiX (X = F, Cl, Br, I).

The Section 2 describes the used quantum chemical methods. The Section 3 tries to determine an accurate value for the Si-H bond length. The Section 4 estimates the accuracy of the ∠(HSiH) and ∠(HSiX) bond angles. The Section 5 is dedicated to the structure of silyl iodide and the Section 6 to the structure of halosilylenes. A general conclusion is provided in Section 7.

## 2. Quantum Chemical Calculations

The structure of the molecules was optimized using three different methods: (i) the second-order Møller–Plesset perturbation theory (MP2) [10], (ii) the hybrid density functional B3LYP [11,12], and (iii) the coupled-cluster method including single and double excitations (CCSD) [13] augmented with a perturbational estimate of the effects of connected triple excitations, CCSD(T) [14]. Several basis sets were used for frozen core calculations: Dunning’s correlation consistent polarized valence triple-zeta basis set: cc-pVTZ for first-row atoms [15], cc-pV(T+d)Z for second-row atoms [16], and for larger atoms, cc-pVTZ-PP with the corresponding small core relativistic pseudopotential [17]. The def2-SVP basis set [18] was also used as well as the 6-311G** basis set [19] for iodine and the 6-311+G(3df, 2pd) [20] for all other atoms.

CCSD(T) calculations correlating all electrons were also performed with the cc-pwCVQZ basis set for first- and second-row atoms [21] and with the cc-pwCVQZ-PP basis set and the corresponding small core pseudopotential [22] for larger atoms.

The CCSD(T) calculations were performed with Molpro [23,24] and the MP2 and B3LYP calculations with Gaussian16 [25].

### Different Types of Structures

The equilibrium structure corresponding to the minimum of the potential hypersurface is obtained by high-level ab initio optimizations. It can also be determined experimentally from a fit of the equilibrium rotational constants (or the corresponding moments of inertia). The equilibrium rotational constants, *B*_e_, are obtained by correcting the ground state rotational constants, *B*_0_, from the rovibrational contribution: $B_{e}=B_{0}+\sum_{i}\alpha_{i}d_{i}/2$ where *α_i_* are the vibration–rotation interaction constants and *d_i_* the degeneracies of these states. The summation is over all the normal modes. The *α_i_* are derived from the rotational constants of the fundamental vibrational states. Alternatively, they can be calculated from an ab initio cubic force field giving the semi-experimental equilibrium structure.

As it is not always easy to determine an equilibrium structure, many empirical methods have been devised.

The simplest one is the effective structure, *r*_0_, which assumes that the equilibrium rotational constants are identical to the ground state rotational constants. Although the rovibrational correction is only a few percent of the rotational constants, it can be a poor approximation of the equilibrium structure.

A slightly better method is the substitution structure, *r*_s_, which assumes that the rovibrational correction is isotopically independent. In this case, the difference between the moment inertia of an isotopologue and the parent species is used.

The *r*_z_ distance is the distance between the average nuclear positions in the vibrational ground state at 0 K. It has a clear physical meaning permitting comparisons between molecules. In this case, it is assumed that the anharmonic part of the rovibrational correction is zero. The *r*_z_ bond length may be used to estimate the equilibrium using the “diatomic approximation” where it is assumed that only the stretching cubic force constant of the bond is different from zero (it is often assumed to be equal to the value of the corresponding diatomic molecule). The accuracy of the *r*_e_-bond lengths derived from the *r*_z_ structure is limited due to the diatomic approximation, and it cannot be better than the accuracy of the *r*_z_ structure, which can be rather poor, in particular, for large molecules. Its accuracy is perhaps not better than a few thousandths of an Å.

## 3. Si-H Bond Length

It is well established that the MP2 method with a basis set of triple zeta quality gives a satisfactory approximation of the C-H bond length [26]. The same conclusion is valid for the Si-H bond, as shown in Table 1 where our ab initio values are compared to the equilibrium values [1,2,3,4,5,27,28,29,30,31,32,33,34,35,36]. 

The median of residuals (*r*_e_–*r*_MP2_) is −0.0008 Å and the mean is −0.0004 Å. The corresponding MAD (mean absolute deviation) is 0.0005 Å corresponding to a standard deviation of 0.0008 Å. In other words, the MP2/cc-pV(T+d)Z level of theory gives a value that is slightly too large (by about 0.0008 Å) but with an excellent accuracy (standard deviation of 0.0008 Å). However, it has to be noted that there are large residuals for most silylene derivatives (up to 0.0095 Å for HSiBr). This discrepancy will be discussed later.

There is another way to estimate the Si-H bond length. It was noticed by Bernstein [43] and developed by McKean [44] that there is a correlation between the isolated C-H stretching frequency (i.e., when only one hydrogen atom is present in the molecule) and the C-H bond length. This correlation permits us to determine the C-H bond length with an accuracy of 0.001 Å [45]. McKean extended this method to the Si-H bond [46,47]; however, to establish the correlation, he used empirical (*r*_0_ or *r*_s_) bond lengths. Using instead equilibrium values, this correlation is confirmed, as shown in Table 1, where most ν_is_(Si–H) values are taken from [6], see also Table 1 for specific references [27,28,32,34,37,38,39,40,41,42]. A fit of 11 data gives the following linear expression:

*r*_e_(Si–H) [Å] = 1.8823(27) − 1.871(12) × 10^−4^*ν*_is_(Si–H) [cm^−1^] (1)

with a correlation coefficient ρ = 0.9997 and a standard deviation σ = 0.0004 Å.

Using Equation (1) and the ab initio results allows us to confirm that the published Si-H bond lengths of most silylene halides are probably inaccurate, see Table 1. This conclusion also applies to dichlorosilane, SiH_2_Cl_2_ [27]. The origin of this discrepancy is easy to explain: the *r*_e_ structures of these molecules were estimated from the *r*_z_ structure and it is known that, in some cases, the method can be inaccurate when the difference *r*_z_–*r*_e_ is large. This is particularly true for the X-H bond lengths. Furthermore, the *r*_z_ structure used for the extrapolation is already not accurate enough.

## 4. (HSiH) Bond Angles

The MP2 method with a basis set of at least triple zeta quality is able to predict the bond angles with an accuracy generally better than 0.4° [48]. This is well verified for the ∠(HSiX) (X = F, Cl, Br, I, CN, SiH_3_, =CH_2_) bond angles as shown in Table 2.

The median as well as the mean of residuals is −0.12°. The corresponding MAD is 0.11° corresponding to a standard deviation of 0.17°, which may be considered as small. Again, the silylene compounds show large deviations. 

## 5. Structure of SiH_3_I 

The method to determine the Si-I bond length is to use a well-conditioned least-squares fit. In such a case, the result is not sensitive to the accuracy of the rovibrational correction as shown for instance in the case of difluoroethane, CH_2_=CF_2_ [49]. One way to obtain a well-conditioned system is to use the method of mixed regression [50]. In this method, structures are fitted concurrently to the moments of inertia and bond parameters obtained from another method. The structures obtained in this work are determined by only three parameters: the Si-H and Si-X bond lengths, and the ∠(HSiX) (or ∠(HSiH)) bond angle. As shown in Section 3 and Section 4, two of these parameters can be determined accurately and can, therefore, be used as predicate observations in the least-squares fit. Everything happens as if there is only one parameter to determine. However, an approximate value of the rovibrational correction is required.

For SiH_3_I, it is relatively easy because it is known that the rovibrational correction *ε* = *I*_0_ − *I*_e_ is approximately proportional to the square root of the moment of inertia [51]. However, empirically, the exponent of *I*_0_ is often found to be different from ½. To determine it, the *ε* of two sets of molecules was used: CH_3_Br and CH_3_I, and SiH_3_Cl and SiH_3_Br. For both sets, an exponent of 0.777 was found. This allowed us to deduce an *ε* value of 0.561 μÅ^2^ for SiH_3_I from that of 0.452 μÅ^2^ for SiH_3_Br. A one-dimensional fit using Si-H = 1.471 Å and ∠(HSiH) = 110.54° gives Si-I = 2.4327 Å. When only one parameter is fitted, the least-squares system is well conditioned (condition number = 1). Therefore, there is no amplification of errors. In this case, ∂*r*(Si-I)/∂*ε* = 0.0082 μ^−1^Å^−1^ to be compared with the value of *ε* which is about 0.66 μÅ^2^. Assuming an error of 0.001 Å for Si-H, 0.3° for ∠(HSiH), and a rather large error of 0.1 μÅ^2^ for *ε*, gives an uncertainty of 0.0012 Å, see Table 3. Using the isotopologues ^28^Si and ^30^Si for the moments of inertia gives identical results. This value is significantly smaller than the *r*_0_ values: 2.437 Å or 2.438 Å. Finally, a mixed regression was performed using three predicate values: *r*(Si-I) = 2.435(5) Å, *r*(Si-H) = 1.470(2) Å; ∠(HSiI) = 108.51(30)° and the three estimated equilibrium *B*_e_ rotational constants of ^28^SiH_3_I, ^2ç^SiH_3_I, and ^30^SiH_3_I. The uncertainty used to weigh these rotational constants was 0.1 MHz. The fit is rather well-conditioned and the residuals are small. The used semi-experimental rotational constants and the residuals of the fit are given in Table S1 of the Supplementary Material. However, the number of data is small: three rotational constants and three predicate values. Therefore, the resulting standard deviations are probably too small.

## 6. Structure of Halosilylenes

For the Si-H bond lengths, the equilibrium values for SiH_2_ [28] and SiHCl [29] are in good agreement with the ab initio optimizations and with the prediction using the isolated stretching frequency, see Table 1 and Table 2. However, for SiHF [30], SiHBr [31], and SiHI [8], there are significant discrepancies. It is important to determine whether these discrepancies are due either to the different bonding or to the poor accuracy of the equilibrium structures. The first information is obtained by comparing the ab initio optimizations and the prediction from the isolated stretching frequencies which are in perfect agreement with the Si-H bond length. However, a more stringent test is welcomed. For this reason, the structures of SiHF, SiHBr, and SiHI have been redetermined.

### 6.1. Structure of SiHF

The equilibrium structure of SiHF has been determined twice [30,52]. In each case, it has been derived from the average (*r*_z_) structure. First, it has to be noted that the Si-H bond length is extremely imprecise and quite different from the predictions of Table 1.

There is an obvious explanation for this poor result. As the molecule is planar, the number of independent rotational constants is only four for SiHF and SiDF whereas there are three parameters to determine. In other words, the system is ill-conditioned and quite sensitive to small errors in the data.

This a typical example where the mixed regression should improve the situation. To calculate a semi-experimental equilibrium structure, the rovibrational corrections were obtained from an ab initio anharmonic force field calculated at the MP2/6-311+G(3df,2pd) level of theory. The ground state rotational constants were taken from Refs. [30,52] and the semi-experimental rotational constants were corrected for a small electronic effect. The values of the diagonal elements of the rotational g-tensor were calculated at the B3LYP/6-311+G(3df,2pd) level of theory. The results are *g_aa_* = −4.29; *g_bb_* = −0.099; *g_cc_* = −1.70. The electronic correction is small for *B* and *C*, of the order of magnitude of one MHz, i.e., similar to the experimental accuracy. On the other hand, it is extremely large for the *A* rotational constants: 534 MHz for SiHF. However, this is not a problem because the *A* rotational constants were not used in the fit. Three predicate values were used: *r*(Si-H) = 1.523(2) Å, *r*(Si-F) = 1.602(5) Å, and ∠(HSiF) = 96.9(5)° (CCSD(T)/cc-pwCVQZ values, all electrons correlated). The estimates of the standard deviations are larger than their probable values to avoid a preponderant role of the predicate values. The predicate value of the Si-F bond length was estimated assuming that the error of the MP2 method is constant for a given bond. In other words:

*r*_e_(Si-F[SiHF]) = *r*_MP2_(Si-F[SiHF) + *r*_e_(Si-F[SiH_3_F]) − *r*_MP2_(Si-F[SiH_3_F]) (2)

The resulting structure is given in Table 4 and the semi-experimental rotational constants and their residuals in Table S2 of the Supplementary Material. From the residuals and the standard deviations of the fitted parameters, it appears that the predicate values are compatible with the semi-experimental rotational constants. In other words, the structure is expected to be reliable.

As the number of data is quite small, the standard deviations of the parameters are not expected to be a reliable estimate of their accuracy. To palliate this inconvenient, the standard deviations were also calculated using the law of propagation of errors assuming an error of 2 MHz for the rotational constants and for the predicates: 0.002 Å for *r*(Si-H), 0.005 Å for *r*(Si-F) and 0.5° for ∠HSiF).

It is interesting to note that the Si-F bond length at 1.603 Å is close to the value found for the radical SiF, 1.601 Å [53].

### 6.2. Structure of SiHCl

There are already equilibrium structures available for SiHCl. The equilibrium structure was first derived from the average (*r*_z_) structure [54]. There are also two high-level ab initio structures [29,55] optimized at the CCSD(T) level of theory. Finally, there are two semi-experimental structures [29] using two different force fields [29], see Table 5. However, there are small differences between these two semi-experimental structures. It might be explained by the fact that rotational constants are available for only three isotopologues, i.e., six independent data (as the molecule is planar). To improve the accuracy, we have repeated the semi-experimental determination by adding the three CCSD(T)_ae/cc-pwCVQZ parameters with conservative uncertainties as predicates, see Table 5. This new structure is in agreement with the previous ones, but it is believed to be slightly more accurate.

### 6.3. Structure of SiHBr

The structure of SiHBr was first determined in 1964 by Herzberg and Varma [56]. They recorded the spectrum of SiHBr in the region 6000 to 4100 Å obtained in the flash photolysis of SiH_3_Br. More recently, Harjanto et al. [42] examined the ground and first excited singlet electronic states of SiHBr through analysis of the 500–400 nm band system, using pulsed discharge jet and laser-induced fluorescence techniques. Rotational analysis of the electronic ground state bands of SiHBr and SiDBr yielded average ground state rotational constants because the bromine isotope splittings could not be resolved. A structural fit using the average of the two bromine isotopologues yielded an effective (*r*_0_) structure. Hostutler et al. [31] determined the ground state harmonic frequencies of gas phase H/DSi^79^Br by exciting single vibronic bands of the $\tilde{A}$^1^A″–$\tilde{X}$^1^A′ electronic transition and recorded the dispersed fluorescence. The derived harmonic force constants and the ground state rotational constants of Harjanto et al. [42] were used to calculate an average (*r*_z_) structure which permitted us to estimate an equilibrium (*r*_e_) structure. Finally, Tackett et al. [57] measured the microwave spectrum by Fourier transform microwave spectroscopy and were able to determine the ground state values of $\bar{B}$ = *B* + *C* for the ^79^Br and ^81^Br isotopologues. We used these values to calculate a semi-experimental equilibrium structure. The rovibrational corrections were obtained from an ab initio anharmonic force field calculated at the MP2/6-311+G(3df,2pd) level of theory. As a check, the calculation was repeated with the def2-TZVP basis set. Almost identical values were obtained. The two experimental data are not enough to determine a complete structure. A sensitivity analysis was first performed and it was found that $\bar{B}$ is mainly sensitive to the value of the *r*(Si-Br) bond length: ∂$\bar{B}$/∂*r*(Si-Br) = 4168 MHz Å^−1^, ∂$\bar{B}$/∂*r*(Si-H) = 80 MHz Å^−1^ and ∂$\bar{B}$/∂(∠SiHBr) = −4 MHz degree^−1^. For this reason, the Si-H bond length derived from the isolated Si-H stretching frequencies (see Table 1) was fixed as well as the ∠(SiHBr) bond angle calculated at the MP2 level of theory (see Table 2). Only the SiBr bond length was fitted. To estimate the accuracy of the result, it was assumed that the standard deviation of *r*(Si-H) is 0.002 Å, that of (∠SiHBr) was 0.4° (in agreement with the results of Section 3 and Section 4), and that the accuracy of the rovibrational correction is 10% [58]. It gives *r*_e_(Si-Br) = 2.2321(4) Å. The structures are given in Table 6.

As a check the Si-Br bond length was estimated from the MP2 optimizations, see Equation (2). It gives *r*(Si-Br) 2.230 Å, in excellent agreement with the previous result.

### 6.4. Structure of SiHI

The analysis of single vibronic level emission spectra of jet-cooled SiHI and SiDI permitted us to determine a harmonic force field [8]. Using this force field and previously determined ground state rotational constants [32], Tackett and Clouthier were able to determine an average (*r*_z_) structure which was used to derive an approximate equilibrium structure. Later, the accuracy of the rotational constants was improved by measuring the Fourier transform microwave spectra of SiHI and SiDI and a new effective structure was determined [9].

To determine a semi-experimental structure, an anharmonic force field was calculated at the MP2/6-311G** level of theory. This permitted us to estimate the rovibrational corrections that were used to correct the ground state rotational constants of Kang et al. [9] and obtain semi-experimental equilibrium moments of inertia. These moments of inertia were fitted by the mixed regression method. Three predicate values were used: (i) the Si-H bond length derived from the isolated Si-H stretching frequencies (see Table 1), (ii) the ∠(HSiI) bond angle calculated at the MP2 level of theory (see Table 2), (iii) an estimate of the Si-I bond length which was estimated assuming that the error of the MP2 method is constant for a given bond, see Equation (2) where F is replaced by I.

As a check, the structure was calculated with anharmonic force fields computed at the MP2/def2-TZVP and B3LYP/def2-TZVP levels of theory. Nearly identical results were obtained. The results are given in Table 7.

## 7. Discussion

Accurate equilibrium structures were determined for SiH_3_I, SiHF, SiHBr, and SiHI. The method was to use well-conditioned least-squares fits, i.e., not sensitive to the uncertainties of the input data. For all these molecules, the structure is defined by three parameters: the *r*(Si-H) and *r*(Si-X) bond lengths, as well as the ∠(HSiX) bond angle (with X = F, Cl, Br, I). It was shown that it is possible to obtain an accurate value of the *r*(Si-H) bond length using either isolated stretching frequencies or MP2 calculations with a triple-zeta basis set. Furthermore, the ∠(HSiX) bond angle can be accurately estimated with MP2 calculations. Finally, using the method of mixed estimation, the third parameter, *r*(Si-X), is determined by the semi-experimental equilibrium moments of inertia. In such a case, it was confirmed that the results are not much sensitive to the accuracy of the rovibrational corrections.

**Figure 1:**
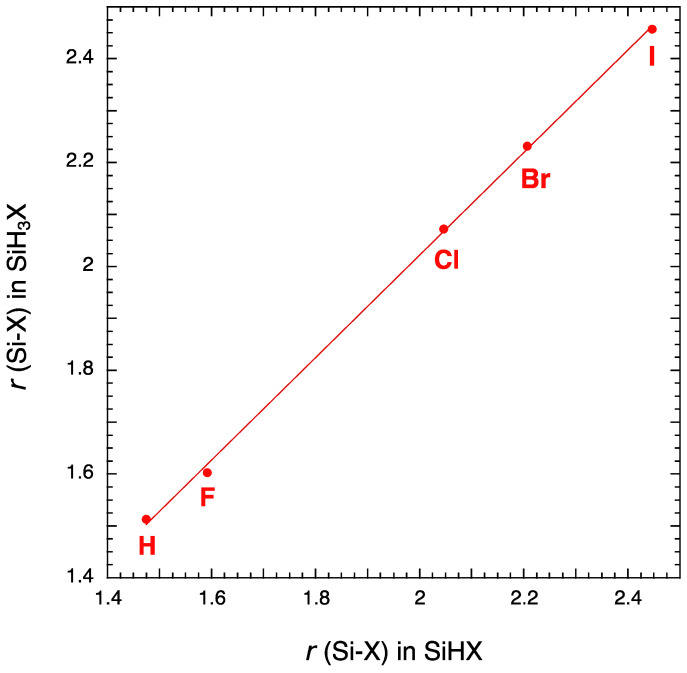
Plot of *r*(Si-X) in SiH_3_X as a function of *r*(Si-X) in SiHX.

There is another way to check the accuracy of the results by plotting the *r*(Si-X) bond of SiHX molecules as a function of the *r*(Si-X) bond of SiH_3_X molecules [59]. As shown in Figure 1, a nice linear correlation is obtained.

Our predictions are also found to be in good agreement with the results of high-level CCSD(T)/cc-pwCVQZ calculations correlating with all electrons (see Table 8). The mean absolute deviation is of 0.0006 and 0.0013 Å for the *r*(Si-H) and *r*(Si-X) bond lengths, respectively, and 0.13° for the HSiX bond angle. 

As already observed by Duncan et al. [6], the structures of the halides form a remarkably consistent set, Si-H bond length and the ∠(HSiX) bond angle remaining almost constant for the silyl halides. For the silylene halides, this conclusion remains true if we exclude SiHF. For the silylene halides, both the bond angle ∠(HSiX) and the bond length *r*(Si-H) increase with the electronegativity of the halogen X.

## Figures and Tables

**Table 1:** Equilibrium values for the Si-H bond length (in Å).

	*r*_e_	MP2 ^a^	*r*_e_—MP2	*ν*_is_ ^b^	Calc ^c^	*r*_e_—calc	Refs. ^d^
SiHD_3_	1.4742	1.4746	−0.0004	2182	1.4740	0.0002	[5], [37]
SiH_3_F	1.4695	1.4703	−0.0008	2207.4	1.4692	0.0003	[1], [6]
SiH_3_Cl	1.4688	1.4698	−0.0010	2206.7	1.4693	−0.0006	[2], [6]
SiH_3_Br	1.4696	1.4699	−0.0003	2205.61	1.4695	0.0001	[3], [6]
SiH_3_CN	1.468	1.4671	0.0009	2217.4	1.4673	0.0007	[4], [38]
SiH_2_F_2_	1.4615	1.4625	−0.0010	2248	1.4616	−0.0001	[33], [39]
SiHF_3_	1.4487	1.4503	−0.0016	2316.9	1.4487	0.0000	[34]
SiH_2_	1.513	1.5115	0.0015	1973.3	1.5130	0.0000	[28], [40]
SiHCl	1.5140	1.5137	0.0003	1968.7	1.5139	0.0001	[29], [28]
SiH_3_SiH_3_	1.4769	1.4779	−0.0010	2162.6	1.4776	−0.0007	[35], [6]
CH_2_=SiH_2_	1.4671	1.4685	−0.0014				[36]
Mediane			−0.0008			0.0000	
Mean			−0.0004			0.0000	
MAD			0.0005			0.0002	
*s*(MAD)			0.0008			0.0003	
**Predictions** ^e^							
SiH_2_Cl_2_	1.4671	1.4643	0.0028	2231.1	1.4648	0.0023	[27], [27]
CH_3_SiH_3_		1.4780		2166.6	1.4768		-, [6]
SiHF	1.529	1.5230	0.006	1930	1.5211	0.008	[30], [41]
SiHBr	1.503	1.5126	−0.010	1976	1.5125	−0.009	[31], [42]
SiHI	1.5151	1.5114	0.0037	1983	1.5112	0.0039	[32], [8]
SiH_3_I		1.4704		2201.1	1.4704		-, [6]

^a^ This work, cc-pVTZ on H, C, N, cc-p(V+d)Z on Si, Cl, cc-pVTZ-PP on Br, I. ^b^ Isolated stretching frequency for the Si-H bond; partially deuterated compounds are used when Si is bonded to more than one H atom. ^c^ Calculated from the isolated stretching frequency. See text. ^d^ The first number gives the reference for *r*_e_ and the second one, the reference for the isolated stretching frequency for the Si-H bond. ^e^ Whereas there are published values for most molecules listed below, they are not accurate as can be seen by comparing with columns MP2 and calc.

**Table 2:** Equilibrium ∠(HSiH) and ∠(HSiX) bond angles in degrees (X = F. Cl. Br. I. …).

	Experimental			MP2/cc-pV(T+d)Z ^a^		
Molecule	HSiH	HSiX	Ref.	HSiH	HSiX	Exp–Calc
SiH_3_F	110.63	108.28	[1]	110.44	108.48	−0.20
SiH_3_Cl	110.47	108.46	[2]	110.39	108.53	−0.07
SiH_3_Br	110.54	108.38	[3]	110.43	108.50	−0.12
SiH_2_F_2_	114.39	108.61	[33]	114.07	108.60	0.01
SiH_2_Cl_2_	112.45	108.67	[27]	112.52	108.56	0.11
SiHF_3_		110.69	[34]		110.49	0.19
SiH_2_	92.04		[28]	92.17	92.17	−0.13
SiH_3_CN	111.43	107.43	[4]	111.23	107.65	−0.22
SiH_3_SiH_3_	115.22	110.23	[35]	108.70	110.24	−0.01
SiHCl		94.66	[29]		95.23	−0.57
CH_2_=SiH_2_	115.22	122.39	[36]	114.51	122.75	−0.36
Mediane						−0.12
Mean						−0.12
MAD						0.11
*s*(MAD)						0.17
**Predictions**						
CH_3_SiH_3_	110.563	108.357	[6]	110.56	108.36	−2.21
SiHF		92.8	[30]		96.90	−4.10
SiHBr		96.9	[31]		94.70	2.20
SiHI		92.5	[8]		93.77	−1.27
SiH_3_I				110.42	108.51	

^a^ cc-pVTZ-PP on Br and I.

**Table 3:** Structure of SiH_3_I (bond lengths in Å, angles in degrees).

Method	*r*(Si-H)	*r*(Si-I)	∠(HSiI)	Ref.
*r*_0_	1.487(8)	2.437(3)	108.4	[7]
*r*_0_	1.474(1)	2.4384(6)	108.16(17)	[6]
MP2 ^a^	1.4704	2.4352	108.507	This work
*r*_e_	1.471 ^b^	2.4327(12)	108.51 ^b^	This work
*r*_e_ ^c^	1.4700(7)	2.4325(2)	108.49(8)	This work

^a^ cc-pVTZ on H, cc-p(V+d)Z on Si, cc-pVTZ-PP on I. ^b^ Fixed value, see text. ^c^ Mixed regression with *r*(Si-I) = 2.435(5) Å, *r*(Si-H) = 1.470(2) Å; ∠(HSiI) = 108.51(30)° and an uncertainty of 0.1 MHz on the *B* rotational constants.

**Table 4:** Structure of HSiF (bond lengths in Å, angle in degrees).

Method	*r*(Si-H)	*r*(Si-F)	∠(HSiF)	Refs.
$r_{e}^{z}$ ^a^	1.528(5)	1.603(3)	96.9(5)	[52]
*r*_e_ ^a^	1.529(6)	1.603(1)	96.9(3)	[30]
MP2/6-311+G(3df,2pd)	1.5201	1.6250	96.59	This work
MP2/cc-pVTZ ^b^	1.5230	1.6132	96.90	This work
$r_{e}^{SE}$	1.5227(8)	1.6028(2)	96.75(8)	This work

^a^ Derived from the *r*_z_ structure. ^b^ cc-pV(T+d)Z on Si.

**Table 5:** Structure of HSiCl (bond lengths in Å, angle in degrees).

Method	*r*(Si-H)	*r*(Si-Cl)	∠(HSiCl)	Refs.
CCSD(T)_ae/cc-pwCVQZ	1.5138	2.0697	95.302	[29]
CCSD(T)_ae/cc-pCVQZ	1.51469	2.07122	95.303	[55]
CCSD(T)_ae/cc-pwCVQZ	1.5138	2.0697	95.31	This work
$r_{e}^{z}$ ^a^	1.525(5)	2.067(3)	96.9(5)	[54]
$r_{e}^{SE}$ ^b^	1.5146	2.0697	94.78	[29]
$r_{e}^{SE}$ ^c^	1.5140	2.0724	94.66	[29]
Predicates ^d^	1.5147(20)	2.0712(50)	95.303(500)	This work
$r_{e}^{SE}$ ^e^	1.5145(7)	2.0719(2)	95.22(11)	This work

^a^ Derived from the *r*_z_ structure. ^b^ Rovibrational corrections from a CCSD(T)/cc-pVTZ cubic force field. ^c^ Rovibrational corrections from a CCSD(T)/cc-pCVTZ cubic force field. ^d^ Predicates from the CCSD(T)/cc-pwCVQZ structure [55]. ^e^ Semi-experimental rotational constants from [29] and predicates from the previous line.

**Table 6:** Structure of HSiBr (bond lengths in Å, angle in degrees).

Method	*r*(Si-H)	*r*(Si-Br)	∠(HSiBr)	Ref.
*r*_0_	1.518(1)	2.237(1)	93.4(3)	[42]
$r_{e}^{z}$ ^a^	1.503(9)	2.235(1)	92.8(4)	[31]
MP2/6-311+G(3df,2pd)	1.5111	2.2439	94.55	This work
MP2/cc-pVTZ ^b^	1.5126	2.2333	94.70	This work
MP2/def2-TZVP	1.5134	2.2390	94.64	This work
$r_{e}^{SE}$	1.5125(20)	2.2321(4)	94.70(40)	This work

^a^ Derived from the *r*_z_ structure. ^b^ cc-pV(T+d)Z on Si, cc-pVTZ-PP on Br.

**Table 7:** Structure of HSiI (bond lengths in Å, angle in degrees).

	*r*(Si-H)	*r*(Si-I)	∠(HSiI)	Refs.
*r*_0_	1.534(1)	2.463(1)	92.4(1)	[8]
$r_{e}^{z}$ ^a^	1.5151(2)	2.4610(1)	92.5(1)	[8]
*r*_0_	1.5405(16)	2.46143(9)	92.68(6)	[9]
MP2/6-311G**	1.5084	2.4967	93.70	This work
MP2/cc-pVTZ ^b^	1.5114	2.4567	93.77	This work
B3LYP/def2-TZVP	1.5230	2.4906	93.41	This work
MP2/def2-TZVP	1.5128	2.4495	93.90	This work
Predicates	1.5112(40) ^c^	2.455(5)	93.8(5) ^d^	This work
$r_{e}^{SE}$ ^e^	1.5138(8)	2.45746(6)	93.26(5)	This work
$r_{e}^{SE}$ ^f^	1.5128(11)	2.45684(8)	93.254(7)	This work
$r_{e}^{SE}$ ^g^	1.5125(7)	2.45760(5)	93.185(4)	This work

^a^ Derived from the *r*_z_ structure. ^b^ cc-pV(T+d)Z on Si and cc-pVTZ-PP on I. ^c^ From isolated stretching frequency, see text. ^d^ MP2 value, see Table 2. ^e^ Cubic force field calculated at the MP2/6-311G** level of theory. ^f^ Cubic force field calculated at the B3LYP/def2-TZVP level of theory. ^g^ Cubic force field calculated at the MP2/def2-TZVP level of theory.

**Table 8:** Comparison of CCSD(T)^a^ and equilibrium structures (bond lengths in Å, angles in degrees).

Molecule	Parameter	CCSD(T) ^a^	*r*_e_ ^b^	*r*_e_—CCSD(T)	Ref. ^c^
HSiF	*r*(Si-H)	1.5235	1.5227	0.0008	This work
	*r*(Si-F)	1.6020	1.6028	−0.0008	
	∠(HSiF)	96.96	96.75	0.21	
HSiCl	*r*(Si-H)	1.5138	1.5145	−0.0007	This work
	*r*(Si-Cl)	2.0697	2.0719	−0.0022	
	∠(HSiCl)	95.31	95.22	0.09	
HSiBr	*r*(Si-H)	1.5127	1.5125	0.0002	This work
	*r*(Si-Br)	2.2323	2.2321	0.0002	
	∠(HSiBr)	94.66	94.70	−0.04	
HSiI	*r*(Si-H)	1.5117	1.5128	−0.0011	This work
	*r*(Si-I)	2.4547	2.4568	−0.0021	
	∠(HSiI)	93.65	93.25	0.40	
SiH_3_F	*r*(Si-H)	1.4694	1.4695	−0.0001	[1]
	*r*(Si-F)	1.5903	1.5915	−0.0012	
	∠(HSiF)	108.41	108.28	0.13	
SiH_3_Cl	*r*(Si-H)	1.4686	1.4688	−0.0002	[2]
	*r*(Si-Cl)	2.0469	2.0458	0.0011	
	∠(HSiCl)	108.52	108.46	0.06	
SiH_3_Br	*r*(Si-H)	1.4687	1.4696	−0.0009	[3]
	*r*(Si-Br)	2.2080	2.207	0.001	
	∠(HSiBr)	108.43	108.38	0.05	
SiH_3_I	*r*(Si-H)	1.4693	1.4700	−0.0007	This work
	*r*(Si-I)	2.4312	2.4325	−0.0013	
	∠(HSiI)	108.46	108.49	−0.03	

^a^ cc-pVQZ on H, cc-pwCVQZ on Si, Cl, cc-pwCVQZ-PP on Br, I, all electrons correlated. ^b^ Experimental or semi-experimental equilibrium structure. ^c^ Reference for *r*_e_.

## Data Availability

The data presented in this study are available in article and Supplementary Materials.

## Supplementary Materials

The following supporting information can be downloaded at: https://www.mdpi.com/article/10.3390/molecules29133101/s1. Table S1. Semi-experimental values for the *B* rotational constants of SiH_3_I and residuals of the fit (MHz). Table S2. Semi-experimental values for the rotational constants of HSiF and residuals of the fit (MHz).
