# Supplementary material for: Equilibrium Values for the Si-H Bond Length and Equilibrium Structures of Silyl Iodide and Halosilylenes
Source: Molecules. 2024 Jun 28;29(13):3101. doi: 10.3390/molecules29133101 (PMC11243611; doi:10.3390/molecules29133101)
Supplement: Supplementary file 1 [file molecules-29-03101-s001.zip › molecules-3059318-supplementary.pdf]

Table S1. Semiexperimental values for the *B* rotational constants of SiH_3I and residuals of the fit (MHz).

Isotopologue	*B*(se)	Exp. – Calc.
$^{28}SiH_3F$	3229.106	0.039
$^{29}SiH_3F$	3153.211	-0.056
$^{30}SiH_3F$	3082.048	0.061

Table S2. Semiexperimental values for the rotational constants of HSiF and residuals of the fit (MHz).

Isotopologue		Constant	Exp. – Calc.
HSiF	*A*	229033(5000)	-639
	B	16964(4)	5.6
	C	15789(4)	-3.7
DSiF	*A*	120362(1000)	-180
	B	16509.7(20)	2.1
	C	14521.3(30)	2.0
